# A Multi-Site Study on Knowledge, Attitudes, Beliefs and Practice of Child-Dog Interactions in Rural China

**DOI:** 10.3390/ijerph10030950

**Published:** 2013-03-07

**Authors:** Jiabin Shen, Shaohua Li, Huiyun Xiang, Shulan Pang, Guozhang Xu, David C. Schwebel

**Affiliations:** 1 Department of Psychology, University of Alabama at Birmingham, 1300 University Blvd., CH 415, Birmingham, AL 35294, USA; 2 School of Public Health Management, Anhui Medical University, 81 Meishan Road, Hefei, Anhui 230032, China; E-Mail: lishaohua168@hotmail.com; 3 Center for Injury Research and Policy, The Research Institute at Nationwide Children’s Hospital, 700 Children’s Drive, Columbus, OH 43205, USA; E-Mail: Huiyun.Xiang@nationwidechildrens.org; 4 School of Public Health, Hebei United University, 46 West Xinhua Road, Tangshan, Hebei 063009, China; E-Mail: pangshu_lan@263.net; 5 Ningbo Municipal Center for Disease Control and Prevention, 237 Yongfeng Road, Ningbo, Zhejiang 315010, China; E-Mail: xugz@nbcdc.org.cn

**Keywords:** dog bites, injury, safety, China, rural health

## Abstract

This study examines demographic, cognitive and behavioral factors that predict pediatric dog-bite injury risk in rural China. A total of 1,537 children (grades 4–6) in rural regions of Anhui, Hebei and Zhejiang Provinces, China completed self-report questionnaires assessing beliefs about and behaviors with dogs. The results showed that almost 30% of children reported a history of dog bites. Children answered 56% of dog-safety knowledge items correctly. Regressions revealed both demographic and cognitive/behavioral factors predicted children’s risky interactions with dogs and dog-bite history. Boys behaved more riskily with dogs and were more frequently bitten. Older children reported greater risks with dogs and more bites. With demographics controlled, attitudes/beliefs of invulnerability, exposure frequency, and dog ownership predicted children’s self-reported risky practice with dogs. Attitudes/beliefs of invulnerability, dog exposure, and dog ownership predicted dog bites. In conclusion, both demographic and cognitive/behavioral factors influenced rural Chinese children’s dog-bite injury risk. Theory-based, empirically-supported intervention programs might reduce dog-bite injuries in rural China.

## 1. Introduction

Dog-bite injury is one of the most common unintentional injuries to children worldwide, both in developed [1,2,3] and developing countries [4,5,6]. Age, gender, and environmental exposure (e.g., dog ownership in the family) are among the most-cited risk factors for dog-bite injuries across cultures [7,8,9], with male children under the age of 14 years with dogs in the home the most common victims of dog-bite injuries globally [6,7,10,11]. In most cultures, rural children are at greater risk of animal-related injuries (including dog-bite injuries) than urban children due to the higher rates of dog ownership and exposure in rural areas [12,13].

Accurate data quantifying dog-bite injuries in China are lacking. Pilot epidemiology suggests animal bites are the third-leading cause of agricultural injuries among rural Chinese, with particularly high rates for rural children [13,14], but no published data focus specifically on dog-bite risk in China. Epidemiologists in Taiwan have found young children were among the most vulnerable populations for dog bites [8]. Researchers in Hong Kong reported similar results, with an average age of dog-bite victims of 11.82 years old [15]. Furthermore, when children experience dog bites, they are more likely to be seriously injured than adults, as indicated by findings in Hong Kong and elsewhere that dogs tend to attack the childrens’ head and neck while adults are injured more frequently on their arms and legs [11,12,15,16,17].

As in much of the world, the risk and significance of pediatric dog-bite injuries is likely greater in rural China than in urban cities for several reasons. First, as a country heavily dependent on agriculture, China has a rural population of over 800 million people, including about 150 million children under age 14 living in rural areas. Children in rural China suffer from the same developmental disadvantages that increase dog-bite vulnerability to children around the world. Compared with adults, children are physically and psychologically underdeveloped [18]. When they encounter dogs, children’s short stature, poor information processing capacity, poor judgment of risk, and immature executive functions endanger them [19]. This threat is substantiated by findings that children are more likely to be bitten by dogs than adults both in developed nations such as the US and in lower income countries like South Africa and Trinidad [4,7,9,20,21]. Second, there are large numbers of dogs in rural China, and thus high exposure opportunity. Unlike urban areas in China where dogs are primarily kept as pets and leashed out of the home [22], dogs are raised in rural China for protection and left to wander the streets unleashed. Protection is deemed important because many adults (especially men) leave rural villages to seek work in larger cities. Dogs are raised to protect the women, elderly and children who remain at home in the rural areas [23]. These dogs escape fences and wander rural areas, posing threat [24,25]. Third, many dogs in rural China have rabies [26,27,28], a factor which substantially increases bite risks to human health and also makes animals more likely to bite. From 1996 to 2006, China experienced a rabies incidence increase of about 2,000%, and the rate has remained stable since [26,27]. Almost all rabies cases in China are transmitted via dog bites [26].

The most commonly practiced measure to prevent dog bites globally is educational campaigns which impart knowledge concerning safe behavior with dogs via live or electronic programs. Empirical research on such campaigns reported mixed success in early evaluations, many of them finding improved knowledge but not safer behaviors [29]. An alternative approach is one that addresses not only an increase in safety knowledge but also considers two other relevant dimensions of cognition, attitudes and beliefs [30]. The Knowledge-Attitudes-Beliefs-Practices (KABP) strategy is recommended by public health specialists to reveal a more complete picture of risk factors underlying health behaviors [31,32,33], and provides valuable information for intervention designed to not only improve knowledge but also translate the knowledge to safer practice of the health behavior.

This study used a culturally-sensitive KABP questionnaire to evaluate knowledge, attitudes, beliefs, and practices of dog safety among children in rural China. Beyond examining descriptive data concerning knowledge, attitudes, beliefs, practices, and self-reported bite history of the sample, we asked five questions: (1) Do children act more riskily with dogs as they grow older? (2) Do boys act more riskily with dogs than girls? (3) Do children who have more frequent contact with dogs, including current or previous ownership of dogs, act more riskily with dogs? (4) Do children with less knowledge about dog safety act more riskily with dogs? and (5) Do children with beliefs/attitudes of invulnerability from dog bites act more riskily with dogs?

## 2. Methods

### 2.1. Participants

A total of 1,537 children (M = 11.26 years old, SD = 1.29) were recruited from the largest primary school in three rural towns: Baishan Town in Hefei City (Anhui Province, *N* = 441), Luanzhou Town in Tangshan City (Hebei Province, *N* = 569) and Hongtang Town in Ningbo City (Zhejiang Province, *N* = 527). All children in grades 4 (age range: 9–11 years old), 5 (age range: 10–12 years old), and 6 (age range: 11–13 years old) in those schools were invited to participate. Each school had three classes in each grade, with class sizes ranging from 50 to 60 students per class. About 95% of children in each classroom participated. The sample was 55% male and 45% female. 96% of the sample self-reported Han ethnicity, with the remaining 4% identifying with one of several minority ethnic groups present in China.

The study protocol was approved by IRB panels at both University of Alabama at Birmingham (USA) and Anhui Medical University (China). The protocol number from the IRB panel at University of Alabama at Birmingham is X120109007; Anhui Medical University does not provide protocol numbers, but the approval is dated 24 December 2011. Written informed consent was obtained from participating children and their parents/legal guardians, as well as from principals of participating schools.

### 2.2. Measures

A Knowledge-Attitudes-Beliefs-Practice (KABP) Questionnaire on child-dog interactions was developed by the authors using the following six steps: (a) thorough review of scientific literature and internet (using structured search strategies) for appropriate content areas on child-dog interaction safety, (b) preparation of items in English by principal investigator (Shen), (c) expert review, face validity review, and editing of items by two senior members of research team, one of them native Chinese (Xiang) and the other familiar with Chinese culture (Schwebel), (d) translation and back-translation from English to Chinese by social scientists fluent in both languages (small differences in translation were resolved through discussion), (e) expert review, face validity review, and editing of items by senior researcher based in China and immersed in culture (Li), and (f) final review and approval by primary investigators (Shen, Schwebel, Xiang).

The questionnaire included four scaled scores: knowledge, attitudes/beliefs, practice, and exposure risk, plus a single item concerning dog bite history. The knowledge scale consisted of 23 questions concerning safe ways to engage with dogs. It was scored as percentile correct, ranging from 0 to 100, with higher scores indicating better mastery of safety knowledge. An example item is as follows: *The Lees are going shopping in the grocery close to their home. They have a child the same age as you are. Their family also keeps a dog that is very friendly. Which of the following choices do you think the Lees should not make? (a) The Lees should leave their child at home and let the dog guard the child and the house; (b) The Lees should take their child with them and let the dog guard the house; (c) The Lees should take both the child and dog with them and lock the house; or (d) The Lees should let one parent stay at home with the child and dog, and let the other parent go shopping.*

The attitudes/beliefs scale consisted of 12 items answered on 5-point scales. The items addressed children’s attitudes and beliefs of invulnerability toward child-dog interactions. An average of responses to the 12 items was used for analysis (range = 1–5), with higher scores indicating riskier attitudes and beliefs and therefore greater levels of perceived invulnerability toward child-dog interactions. Example items are “*I think a small scratch from dog bite does not need going to hospital.*” and “*I think the dog in my own family is less likely to bite people than the average dog.*”

The practice scale consisted of 8 items answered on 5-point scales (range = 1–5), with higher scores indicating higher frequency of self-reported risky behavior with dogs. Example items are “*Pet a sleeping dog*” and “*Play with puppy dogs when their mother is present*”.

The exposure scale consisted of a single item, “*How often do you usually interact with dogs?*” Children chose the response that is closest to their situation on a 6-point scale ranging from “*Never—I’ve never interacted with a dog*” to “*At least once a day*”. Higher scores indicate more frequent contact with dogs. The dog bite history item assessed self-reported frequency of actual dog bites, which was dichotomized into lifetime history of no bites *versus* one or more bites.

Psychometrics of relevant scales were strong. Cronbach’s alphas for the attitudes/beliefs and practice sections were 0.77 and 0.76, respectively. We did not compute Cronbach’s alpha for the knowledge section because different domains of safety knowledge on dogs may be theoretically expected to be unrelated to each other. In addition to the other items, children completed brief items concerning their age, gender, and ethnicity.

## 3. Results

Table 1 shows descriptive statistics and intercorrelations between primary variables. 29.7% of children (n = 459) reported a history of being bit by a dog. The mean score for exposure frequency to dogs was 4.59 (SD = 1.59), indicating that children on average interacted with dogs several times a month. 64% of the sample indicated daily or weekly interactions with dogs. The mean score on dog safety knowledge was 55.5% (SD = 14.2%), indicating the children knew just over half the facts presented to them about how to behave safely with dogs. The mean score of attitudes/beliefs of invulnerability was 1.91 (SD = 0.58), suggesting on average the youth had moderately safe attitudes and beliefs about engagement with dogs (2.00 reflected “somewhat disagreeing” with conducting dangerous activities with dogs). The mean score for practice was 1.63 (SD = 0.57), indicating the children behaved relatively safely with dogs in their daily life (1 represented “never” and 2 “occasionally” for conducting dangerous activities with dogs). Assumptions for the inferential statistical analyses were examined and no serious violations were found.

**Table 1:** Descriptive statistics and correlation matrix of age, gender, exposure frequency of exposure, knowledge, attitudes/beliefs, practice and bite history.

Variable	Mean	SD	1	2	3	4	5	6	7
1. Age	11.26	1.29	1.00						
(Years)									
2. Gender	0.55	0.50	0.05	1.00					
(0 = Female, 1 = Male)									
3. Safety knowledge	55.53	14.17	−0.03	−0.07 **	1.00				
(% correct, 0–100 scale)									
4. Attitudes/beliefs	1.91	0.58	0.18 **	0.06 *	−0.43 **	1.00			
(5-point scale, 1–5)									
5. Risky practice	1.63	0.57	0.18 **	0.14 **	−0.23 **	0.47 **	1.00		
(5-point scale, 1–5)									
6. Exposure frequency	4.59	1.59	0.12 **	0.09 **	−0.06 *	0.21 **	0.35 **	1.00	
(6-point scale, 1–6)									
7. Bite history	0.30	0.46	0.07 **	0.10 **	−0.03	0.09 **	0.12 **	0.11 **	1.00
(0 = No, 1 = Yes)									

*p < 0.05; **p < 0.01.

Gender comparisons revealed that boys (M = 4.72, SD = 1.51) had more frequent exposure to dogs than girls (M = 4.43, SD = 1.68), *t* (1, 390) = 3.50, *p* < 0.001. Boys (M = 54.64, SD = 14.47) also scored slightly lower on the knowledge scale than girls (M = 56.59, SD = 13.74), *t* (1, 504) = –2.70, *p* < 0.001. On attitudes/beliefs of invulnerability, boys (M = 1.94, SD = 0.60) scored higher (thus riskier) than girls (M = 1.87, SD = 0.55), *t* (1, 507) = 2.26, *p <* 0.05. Finally, boys (M = 1.70, SD = 0 .59) reported higher (thus riskier) behavioral practices with dogs than girls (M = 1.54, SD = 0.53), *t* (1, 508) = 5.49, *p* < 0.001. The gender differences were reflected also in self-reported bite history, with 34.0% of boys reporting having been bitten by dogs while only 24.8% of girls reported having bite history, χ^2^ (1) = 15.33, *p* < 0.001. Children’s age was significantly correlated with greater exposure to dogs (*r* = 0.12, *p* < 0.001), riskier attitudes/beliefs of invulnerability (*r* = 0.18, *p* < 0.001), riskier behaviors with dogs (*r* = 0.18, *p* < 0.001), and history of dog-bites (*r* = 0.07, *p <* 0.01), but not with safety knowledge.

Relations between knowledge, attitudes/beliefs, practice, and bite history were considered next (see Table 1). Children with more safety knowledge tended to hold fewer attitudes/beliefs of invulnerability toward child-dog interactions (*r* = −0.43, *p* < 0.001), and also reported safer behavior in their daily interaction with dogs (*r* = −0.23, *p* < 0.001). As expected, the attitudes/beliefs score was positively correlated with the risky practice score (*r* = 0.47, *p* < 0.001). Bite history was significantly correlated with attitudes/beliefs of invulnerability (*r* = 0.09, *p* < 0.001) and risky practice (*r* = 0.12, *p* < 0.001) but not safety knowledge (*r* = −0.03).

Among the 1528 children (99.4% of sample) who responded validly concerning dog ownership, 578 (37.8%) reported they currently owned a dog at home, 657 children (43.0%) reported that they once owned a dog but did not have one now, and 293 children (19.2%) reported no history of owning a dog at home. There were no gender or age differences*.*

One-way analysis of variance (ANOVA) compared the three dog-ownership groups on exposure frequency to dogs, safety knowledge, attitudes/beliefs of invulnerability, risky practice with dogs, and dog bite history (see Table 2). There were no significant differences among the three groups in safety knowledge. On attitudes/beliefs of invulnerability, children who had never owned a dog at home (*M* = 1.80, *SD* = 0.51) perceived less vulnerability than both children who owned a dog before but not now (*M* = 1.94, *SD* = 0.56) and children who owned a dog currently (*M* = 1.92, *SD =* 0.63), *F* (2, 1,498) = 6.19, *p* < 0.01. No significant differences were found between the latter two groups in post-hoc tests. Similar results were found concerning children’s self-reported risky practice with dogs, with children who had no history of owning a dog at home reporting safer behavior with dogs (*M* = 1.39, *SD* = 0.42) than children who previously owned a dog but not now (*M* = 1.67, *SD* = 0.54) and children who currently owned a dog at home (*M* = 1.70, *SD* = 0.61), *F* (2, 1,498) = 34.23, *p* < 0.01. On exposure frequency, children who currently owned a dog at home had the highest exposure frequency (*M* = 5.15, *SD* = 1.34), followed by children who previously owned a dog at home but not now (*M* = 4.57, *SD* = 1.49) and children who had never owned a dog (*M* = 3.52, *SD* = 1.73), *F* (2, 1,506) = 114.75, *p* < 0.001.

The final step of analysis was construction of two-step multivariate regression models to evaluate whether exposure frequency, dog-ownership, knowledge and attitudes/beliefs predicted risky practice and/or history of bites, after controlling for demographic factors. Dog ownership was dummy-coded with no history of dog ownership as the referent.

In the models predicting self-reported risky practice with dogs (see Table 3), gender and age were entered first as independent variables and accounted for 5% of the variance in children’s self-reported risky behavior practice with dogs, *R^2^* = 0.05, *F* (2, 1,467)= 40.18, *p* < 0.001. As children grew older, they behaved more riskily with dogs. Boys also behaved more riskily with dogs than girls. The second regression model predicting risky practice with dogs evaluated whether exposure frequency, dog-ownership, safety knowledge, and attitudes/beliefs of invulnerability predicted risky behavior after controlling for demographic factors. Exposure frequency (*t* = 9.75, *p* < 0.001), owning a dog now (*t* = 3.33, *p <* 0.001), owning a dog before but not now (*t* = 3.69, *p* < 0.001), and attitudes/beliefs (*t* = 15.19, *p <* 0.001) but not knowledge (*t* = −1.28), accounting for a significant proportion of the variance, *R^2^* change = 0.26, *F* (5, 1,462) = 111.27, *p* < 0.001. Thus, children acted more riskily with dogs if they had more frequent exposure to dogs, if they had owned a dog before or currently, and if they held riskier attitudes/beliefs of invulnerability toward dogs. They did not act more riskily with dogs if they had less knowledge about dog safety.

**Table 2:** Comparison of exposure frequency, bite history and KABP among different dog-ownership groups.

	Never own a dog	Previously own a dog	Currently own a dog	*F*	*η^2^*
	(n = 293, 19.2%)	(n = 657, 43.0%)	(n = 578, 37.8%)		
Safety knowledge	56.94 (13.53)	55.38 (14.05)	54.98 (14.62)	1.92	0.00
(% correct, 0–100 scale)					
Attitudes/beliefs	1.80 (0.51) ^a,b^	1.94 (0.56) ^a^	1.92 (0.63) ^b^	6.19 **	0.01
(5-point scale, 1–5)					
Risky practice	1.39 (0.42) ^c,d^	1.67 (0.54) ^c^	1.70 (0.61) ^d^	34.23 **	0.04
(5-point scale, 1–5)					
Exposure frequency	3.52 (1.73) ^e^	4.57 (1.49) ^e^	5.15 (1.34) ^e^	114.75 **	0.13
(6-point scale, 1–6)					
Bite history ^1^	4.30% ^f,g^	13.90% ^f^	11.60% ^g^	10.47 **	0.08
(0 = No, 1 = Yes)					

** p < 0.01. ^1^ χ^2^ and Cramer’s V reported for the categorical Bite History variable. ^a ^*p* = 0.002; ^b ^*p* = 0.008; ^c ^*p* = 0.000; ^d ^*p* = 0.000; ^e ^*p* = 0.000; ^f ^*p* = 0.001; ^g ^*p* = 0.009. *Post-hocs* a–e were conducted using Bonferroni test; f and g were conducted using chi-square test.

**Table 3:** Hierarchical linear regression analysis predicting children’s risky practice with dogs.

Variable	Model 1			Model 2		
	*B*	*SE B*	*β*	*B*	*SE B*	*β*
Age (Years)	0.08	0.01	0.18 **	0.04	0.01	0.08 **
Gender (0 = Female, 1 = Male)	−0.15	0.03	0.13 **	−0.10	0.03	0.09 **
Dog ownership						
* (No dog ownership history: referent)*						
* Owning a dog now*	-	-	-	0.12	0.04	0.11 **
* Owning a dog before but not now*	-	-	-	0.13	0.04	0.11 **
Safety knowledge (% correct, 0–100 scale)	-	-	-	−0.00	0.00	−0.03
Attitudes/beliefs (5-point scale, 1–5)	-	-	-	0.37	0.02	0.38 **
Exposure frequency (6-point scale, 1–6)	-	-	-	0.08	0.01	0.27 **
*R^2 ^*change	0.05			0.26		
*df1/df2 for R^2 ^*change	2/1,467			5/1,462		
*F* for *R^2^* change	40.18 **			111.27 **		

** *p <* 0.01

In models predicting dog bite history (see Table 4), the same predictors were entered into a logistic regression. Gender and age were entered in the first model as demographic predictors and were found to contribute significantly to the prediction of children’s dog-bite history, χ^2^ (2) = 21.51, *p* < 0.001. Boys were 1.56 times more likely to be bitten by dogs than girls (*OR* = 1.56, 95%CI = 1.24–1.96). A one-year increase in age was associated with 1.12 times more likely risk for bites (*OR* = 1.12, 95%CI= 1.02–1.22). Knowledge, attitudes/beliefs, exposure frequency and dog-ownership were entered along with age and gender in the second logistic regression model predicting dog-bite history. The full model significantly reduced the *−2 Log Likelihood* of the first model by 22.10, χ^2^ (5) = 22.10, *p* < 0.001. With all predictors included, gender (*OR* = 1.50, 95%CI = 1.19–1.89) was still a significant predictor but age was not. Also significant predictors were higher attitudes/beliefs of invulnerability (*OR* = 1.26, 95%CI = 1.01–1.57), more frequent exposure to dogs (*OR=* 1.11, 95%CI = 1.02–1.20), and previously owning a dog (*OR*= 1.45, 95%CI = 1.03–2.05). Thus, male gender, attitudes/beliefs of invulnerability, more frequent exposure to dogs, and previous but not current ownership of a dog were associated with history of a dog bite. Knowledge about dog safety and current dog ownership was not.

**Table 4:** Hierarchical logistic regression analysis predicting children’s dog-bite history.

Variable	Model 1		Model 2	
	*OR*	*95%CI*	*OR*	*95%CI*
Age (Years)	1.12 *	1.02–1.22	1.08	0.98–1.89
Gender (0 = Female, 1 = Male)	1.56 **	1.24–1.96	1.50 **	1.19–1.89
Dog ownership				
* (No dog ownership history: referent)*				
* Owning a dog now*	-	-	1.28	0.89–1.84
* Owning a dog before but not now*	-	-	1.45 *	1.03–2.05
Safety knowledge (% correct, 0–100 scale)	-	-	1.00	0.99–1.01
Attitudes/beliefs (5-point scale, 1–5)	-	-	1.26 *	1.01–1.57
Exposure frequency (6-point scale, 1–6)	-	-	1.11 *	1.02–1.20

* p < 0.05, ** p < 0.01.

## 4. Discussion

### 4.1. Risk Factors for Pediatric Dog-Bite Injuries in Rural China

Consistent with findings from other countries [7,8,11,12,34], gender and age were significant risk factors for pediatric dog-bite injuries in rural China. Boys reported taking greater risks in interacting with dogs and higher rates of bite injury than girls. As children grew older and approached adolescence, they tended to report riskier attitudes, beliefs about invulnerability to risk with dogs, and self-reported riskier behaviors with dogs, despite the fact that they had similar levels of safety knowledge as the younger counterparts. These children also reported more bites in their history. These findings are particularly concerning since older children may have greater independence in deciding how to act when unsupervised around dogs.

Cognitive/behavioral factors influenced children’s self-reported risky behaviors with dogs. After controlling for age and gender, greater exposure to dogs and perceptions of greater invulnerability to bites were associated with riskier child-dog interactions. We cannot infer causality from this cross-sectional dataset, but one possible explanation is that children with a higher familiarity with dogs perceive dogs as less dangerous and less likely to bite them, and therefore take greater risks with dogs. This hypothesis is supported by findings from college students in the United States [35].

Cognitive/behavioral factors also influenced children’s self-reported history of dog bites. After controlling for age and gender, holding more attitudes/beliefs of invulnerability, having previous experience owning a dog and having more frequent exposure to dogs contributed to children’s self-reported dog bite history. Therefore, it appears that children who held more attitudes/beliefs of invulnerability, and who had experience raising a dog and higher exposure frequency to dogs not only engaged in dangerous interactions with dogs but also were more likely to have experienced dog bites. The significant correlation between dog bite history and risky practice (*r* = 0.12, *p* < 0.01) also supported this conclusion. Current ownership of dogs was not associated with history of dog bites even though previous dog ownership was. It may be that some families whose dogs bite children kill or remove the dog from the home.

### 4.2. Implications for Intervention Development

Several programs aiming to prevent dog-bite injuries among children have been developed and tested globally. Some focus on individual (e.g., child or parent) change and others focus on environmental change. At the individual level, the most promising programs incorporate behavioral strategies to improve children’s knowledge and behavior, either in classroom environments or via computer software [36,37,38,39,40,41]. In general, these programs are effective at helping children learn more about dog safety but show limited evidence of creating behavior change [36,40].

Other programs focus on environmental changes. With collaborative effort from entire communities, these is some evidence that dog bite incidence can be reduced [42]. Both behavioral and community-based environment change strategies may be effective to reduce risk in rural China.

Of course, any interventions in rural China will need to be sensitive to local cultural and contextual issues. On average, children in this study answered only 56% of safety knowledge questions correctly, suggesting pre-teens in rural China have poor knowledge about how to interact safely with dogs, even though stray dogs routinely wander the streets in their communities. Improving children’s knowledge could be one important intervention strategy that ultimately leads to safer behavior and a reduction of pediatric dog bites in rural China. The correlations between knowledge and both attitudes/beliefs and practice support the reasoning that improved knowledge might result in healthier attitudes and beliefs and ultimately in safer practice.

The fact that rural Chinese children with perceived invulnerability to risk reported riskier behavior with dogs and higher rates of bite injuries could also lead to other intervention strategies, including behavioral strategies to increase perceived vulnerability and to change peer norms. Taken together, public health interventions targeting knowledge, attitudes, beliefs, and behavioral change might all be instituted with awareness of the local culture. The Chinese education system supports classroom-based education on both knowledge and attitudes/behavioral skills [43], and intervention programs should be consistent with cultural practices concerning health education.

### 4.3. Limitations and Future Directions

One limitation of this study is that the data were self-reported. Although anonymous, it is possible that children, and perhaps especially older boys, felt proud to showcase their “brave” but risky interactions with dogs, especially given the fact that data were collected in a classroom setting with peers and teachers nearby. Self-reporting was also unable to detect the difference between children’s perceived “dog bite” and a clinically diagnosed dog bite. It also is possible that children’s recall of bite injuries and actual practice with dogs was biased or incorrect. Another limitation is the relatively narrow age range of our sample, with all children recruited from grades 4 to 6. It is possible that the age range was insufficient to detect developmental trends in knowledge, attitudes and/or behaviors with dogs in rural China that occur in younger or older children. Finally, we did not assess for history of exposure to rabies. It may be that children exposed to rabies react differently to dog bites *versus* those exposed to bites from dogs known to be non-rabid.

Future research might take several directions. Behavioral measures (e.g., naturalistic observation of children’s daily interactions with dogs) would be valuable, as would review of medical records to obtain more accurate bite injury history data to compare with children’s self-reported dog bites. Replication of this study with children across a larger age range, or in other regions (both rural and urban) of China, and with children from other low- and middle-income nations would also be valuable.

## 5. Conclusions

This study investigated dog-bite risk among children in grades 4–6 in rural China. Our results suggest that male gender, older age, frequent exposure to dogs, previous ownership of dogs, and attitudes/beliefs of invulnerability are factors that place children at increased risk of dog-bite injuries in rural China. More specifically, boys were more frequently exposed to dogs than girls. They also possessed less safety knowledge but riskier attitudes, beliefs of invulnerability, and more dangerous self-reported practices with dogs than girls. As children grew older, they reported greater exposure to dogs, but they also held riskier attitudes/beliefs and reported more risky behavior practices with dogs. Furthermore, children with riskier attitudes, beliefs of invulnerability, and those who were more frequently exposed to dogs and who previously owned a dog tended to report taking more risks with dogs and to be bitten by dogs more often.
